# Removal of a partial genomic duplication restores synaptic transmission and behavior in the MyosinVA mutant mouse Flailer

**DOI:** 10.1186/s12915-023-01714-y

**Published:** 2023-11-14

**Authors:** Fernando J. Bustos, Swarna Pandian, Henny Haensgen, Jian-Ping Zhao, Haley Strouf, Matthias Heidenreich, Lukasz Swiech, Benjamin E. Deverman, Viviana Gradinaru, Feng Zhang, Martha Constantine-Paton

**Affiliations:** 1 McGovern Institute for Brain Research, Department of Brain and Cognitive Sciences, Massachusetts Institute of Technology, Cambridge, MA USA; 2 Instituto de Ciencias Biomedicas, Facultad de Medicina y Facultad de Ciencias de La Vida, Universidad Andres Bello, Santiago, Chile; 3 Broad Institute of MIT and Harvard, Cambridge, MA USA; 4 Stanley Center for Psychiatric Research, Broad Institute of MIT and Harvard, Cambridge, MA 02142 USA; 5 Division of Biology and Biological Engineering, California Institute of Technology, Pasadena, CA USA; 6 Department of Biological Engineering, Massachusetts Institute of Technology, Cambridge, MA USA; 7 Howard Hughes Medical Institute, Cambridge, MA USA

**Keywords:** DN-CRISPR, ASD, Anxiety, Gene-editing, Therapy

## Abstract

**Background:**

Copy number variations, and particularly duplications of genomic regions, have been strongly associated with various neurodegenerative conditions including autism spectrum disorder (ASD). These genetic variations have been found to have a significant impact on brain development and function, which can lead to the emergence of neurological and behavioral symptoms. Developing strategies to target these genomic duplications has been challenging, as the presence of endogenous copies of the duplicate genes often complicates the editing strategies.

**Results:**

Using the ASD and anxiety mouse model Flailer, which contains a partial genomic duplication working as a dominant negative for MyoVa, we demonstrate the use of DN-CRISPRs to remove a 700 bp genomic region in vitro and in vivo. Importantly, DN-CRISPRs have not been used to remove genomic regions using sgRNA with an offset greater than 300 bp. We found that editing the *flailer* gene in primary cortical neurons reverts synaptic transport and transmission defects. Moreover, long-term depression (LTD), disrupted in Flailer animals, is recovered after gene editing. Delivery of DN-CRISPRs in vivo shows that local delivery to the ventral hippocampus can rescue some of the mutant behaviors, while intracerebroventricular delivery, completely recovers the Flailer animal phenotype associated to anxiety and ASD.

**Conclusions:**

Our results demonstrate the potential of DN-CRISPR to efficiently remove larger genomic duplications, working as a new gene therapy approach for treating neurodegenerative diseases.

**Supplementary Information:**

The online version contains supplementary material available at 10.1186/s12915-023-01714-y.

## Background

Genomic recombination events, and particularly copy number variants, have been strongly linked to neurodegenerative diseases such as Alzheimer’s disease and especially autism spectrum disorders (ASD) [1–6]. Targeting these sequences using gene editing tools has proven challenging, as editing a genomic duplication may also affect the endogenous copies of the genes that have been duplicated. Therefore, identifying new strategies for editing genomic duplications without altering the endogenous copies of the wild-type genes holds promise for future therapeutic approaches.

In recent years, the discovery and development of CRISPR/Cas9 has enabled the versatile manipulation of the genome [7–13]. Among CRISPRs, double-nicking CRISPRs (DN-CRISPRs) have emerged as an alternative to gain on-target specificity in genome editing [14–17]. DN-CRISPRs utilize SpCas9n (Cas9n), a mutated form of SpCas9 (D10A, Cas9) that, instead of producing double-strand breaks, can only nick one DNA strand. This nick can be efficiently repaired by the cell without introducing genetic mutations [14]. To successfully edit the genome, it utilizes two sgRNAs that must be targeted to opposite strands of DNA at the desired genomic locus. Previous studies have shown that when the separation, or offset, between the two sgRNAs is more than 100 bp [14] or, at most, 300 bp [17], excision efficiency diminishes, limiting the use of this approach. Nevertheless, here we demonstrate the feasibility of using DN-CRISPR to remove a larger genomic region using sgRNAs with an offset greater than 700 bp.

**Fig. 1:**
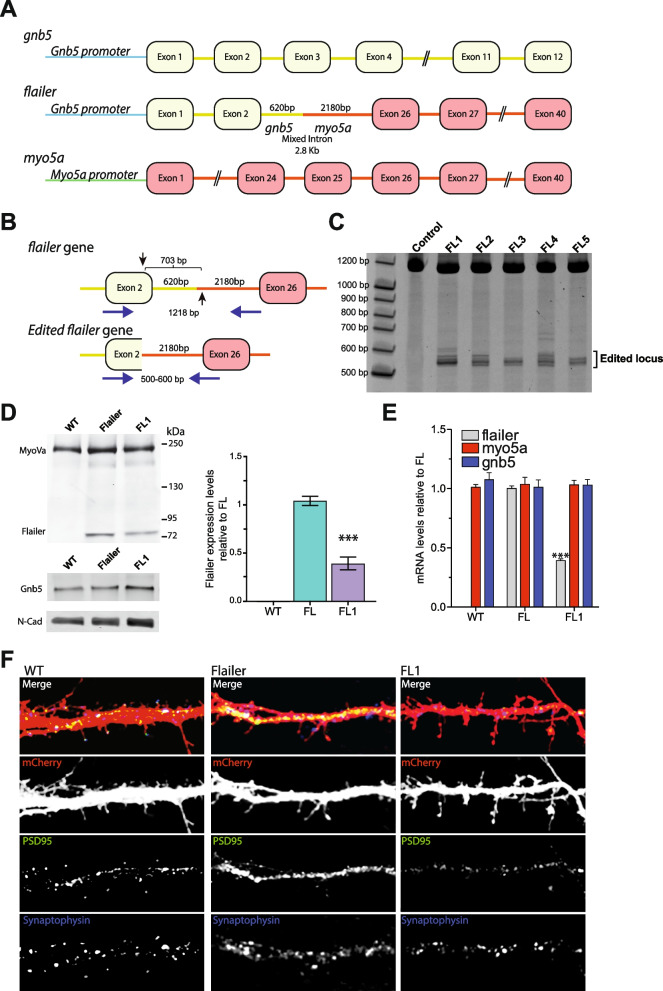
Targeting the Flailer genomic locus with DN-CRISPRs. **A** Scheme of *gnb5*, *flailer*, and myo5a genes. **B** Schematic representation of *flailer* editing. Black arrows indicate the region of the DNA targeted by sgRNAs to edit the *flailer* gene via DN-CRISPRs. sgRNAs for DN-CRISPRs need to be targeted at opposite strands. Red arrows show the primers used for PCR analysis to determine the status of the genomic locus. **C** PCR products to analyze gene editing of flailer-infected neurons. Protein (**D**) and mRNA (**E**) expression levels infected with FL1-FL5. **F** Immunofluorescences against PSD95 and Synaptophysin in wild-type, FL, and FL1-injected neurons. Bars represent mean ± SEM; ***p < 0.001. One-way ANOVA was used to determine significance

The Flailer mouse was identified as a mouse model that carries a spontaneous non-homologous recombination event that resulted in an extra gene, named *flailer*, composed by the fusion of the promoter and exons 1–2 of the *gnb5* gene fused together by a mixed intron, to exons 26–40 of the *myo5a* gene [18] (Fig. 1A). In Flailer animals, cells harbor the endogenous copies of *gnb5*, *myo5a*, and *flailer* genes on chromosome 9 (Fig. 1A)*.* The expression of the Flailer fusion protein is controlled by the gnb5 gene promoter, which is highly and broadly expressed in the central nervous system (CNS) [19, 20]. The resulting protein, Flailer, binds to cargo but lacks the actin-binding domain of MyosinVa, which is necessary for binding and walking to the plus end of actin filaments [21, 22]. In wild-type neurons, MyosinVa plays a crucial role in transporting various components of the synaptic complex, including scaffolding proteins, receptors, and mRNAs, to dendritic spines [23–27]. This process is essential for proper synaptic function and plasticity. When the Flailer protein is present in a 1:1 ratio with wild-type MyosinVa it works as a dominant negative, leading to a reduced transport and abnormal synaptic clustering of receptors and scaffolding proteins such as PSD95 and AMPA receptors [26, 28]. Importantly, removing at least one copy of the Flailer gene could theoretically lead to the recovery of the phenotypes. Previous studies have shown that Flailer neurons exhibit decreased numbers of synaptic spines, increased AMPA receptor-mediated currents, and lack of long-term depression (LTD) in the visual cortex and hippocampus [26, 28]. The enhancement of synaptic activity observed in the FL animal is a result of the absence of LTD. This lack of LTD leads to an inability to prune synapses, resulting in the formation of new synaptic contacts in the dendritic shafts. This is due to the broad distribution of receptors and scaffolds in these regions, as transport to spines is impaired [26]. Furthermore, due to the synaptic defects, Flailer mice display behaviors associated with ASD and anxiety, such as repetitive grooming, seizures, memory deficits, and increased anxiety-like behavior [28]. The Flailer mouse model presents a unique opportunity to dissect the role of different brain regions in controlling behaviors. Since the entire brain of Flailer mice carries the mutation, removing the duplication in specific brain regions allows us to determine the sufficiency of a brain area in controlling a specific behavior and to study its underlying neural circuits.

In this study, we utilized DN-CRISPR technology designing pairs of sgRNAs to target exon 2 of *gnb5* and the *myo5a* mixed intron region of the *flailer* gene, which encompasses 703 bp. By targeting the coding sequence, we aimed to knock out the Flailer gene while also providing the specificity needed to avoid altering the endogenous copies of the genes involved in the partial duplication. We delivered the sgRNAs via AAV in vitro and in vivo. Our results showed a reduction in the expression of the Flailer protein and mRNA, and the rescue of synaptic transmission defects present in Flailer neurons in culture and recovery of LTD in hippocampal slices. Our in vivo experiments targeting the ventral hippocampus, show that removal of *flailer* allows for partial recovery of anxiety behaviors, while systemic brain delivery of DN-CRISPRs was able to fully recover the behavioral phenotypes exhibited by Flailer animals.

Altogether, our findings demonstrate the potential of DN-CRISPRs as a tool for targeting genomic duplications and investigating their effects on neural function and behavior. Moreover, we show that DN-CRISPRs can be used to target large genomic regions with high efficiency, which opens the possibility to new therapeutic strategies.

## Results

### Gene editing of the *flailer* gene by DN-CRISPR

As previously described [18], the *flailer* gene is composed of the first two exons of *gnb5* fused in a frame with a mixed intron (*gnb5/myo5a)* and exons 26–40 of *myo5a* (Fig. 1A). Since the Flailer mutation is a partial duplication, every cell also contains the endogenous copies of gnb5 and myo5a (Fig. 1A) [18]. To specifically edit the *flailer* gene, we employed DN-CRISPRs, as using CRISPR/Cas9 to edit the mutation/duplication would also affect the endogenous copies gnb5 and *myo5a.* If the endogenous copies of either *gnb5* or *moy5a* are targeted, a new mutation would arise producing a different phenotype. Therefore, this must be avoided. It has been reported that for DN-CRISPR to be able to edit the genome, two sgRNAs need to be in proximity (small offset) on opposite strands. Our strategy proved challenging since the offset of sgRNAs to specifically target *flailer* needed to be greater than 700 bp, and as previous studies have demonstrated, when the distance between the two sgRNAs needed to target the genomic locus is greater than 300 bp the efficiency of editing by DN-CRISPRs is limited [14, 17, 29].

Pairs of sgRNAs were designed to target exon 2 of gnb5 and the *myo5a* mixed intron part of the *flailer* gene, a region encompassing approximately 703 bp (Fig. 1B; black arrows). By targeting the coding sequence, we aimed to knock out the *flailer* gene to recover the FL phenotype. To identify the most efficient gRNA pair for *flailer* editing, primary cortical neurons were transduced via AAV delivery at 3 days in vitro (DIV) with TdTomato alone or in combination with Cas9n and five different sgRNA pairs (FL1-FL5). Six days after infection, genomic DNA was extracted and amplified by PCR using specific primers targeting the *flailer* mutated region (Fig. 1B; red arrows). Neurons carrying the complete *flailer* gene are expected to produce a 1218 bp PCR product (Fig. 1B; top-red arrows), while edited Flailer gene sequences produce a 500–600 bp PCR product (Fig. 1B; bottom-red arrows). As expected, control neurons infected only with Cas9n and TdTomato show a single PCR product of 1218 bp, while neurons co-infected with Cas9n and sgRNAs (FL1-5) exhibit a collection of PCR products at ~ 500–600 bp, indicating successful editing of the locus (Fig. 1C). All subsequent experiments were conducted using the FL1 pair of sgRNAs (FL1: *gnb5*: 5′ CTTTGCACCAATCCATGCAC 3′; *myo5a:* 5′ ATGTTCATGCTTCTATTGAC 3′) + Cas9n (henceforth FL1 for simplicity), as it demonstrated the highest efficiency in editing the Flailer genomic locus. Products obtained from the PCR amplification were purified and sequenced to qualitatively assess the indels produced at the targeted location (Additional file 1: Fig. S1A). In addition, locus-specific sequencing (Additional file 1: Fig. S1B–C), and surveyor assays (Additional file 1: Fig. S1D–E) of the endogenous *gnb5* and *myo5a genomic* sequences, that are also nicked by FL1 + Cas9n, revealed that genomic regions were not altered by DN-CRISPR but indel mutations were detected when targeting with wild-type-SpCas9 (Additional file 1: Fig. S1B–E). To determine the impact of gene editing on FL, we quantified mRNA and protein levels. FL neurons infected for 10 days with FL1 show a robust 70% reduction in protein and mRNA levels compared to FL neurons (Fig. 1D–E). As expected, since *gnb5* and *myo5a* genomic locus remained unedited, we did not observe any changes in the mRNA and protein expression levels of wild-type MyoVa or Gnb5 in FL1 treated cells (Fig. 1D–E).

We have previously shown that FL-cultured neurons exhibit impaired transport of proteins to the synapse, resulting in a lack of the characteristic clustering structure of receptors and scaffolding proteins such as PSD95 and AMPA receptors. As a result, receptors are distributed extensively in the dendrite shafts where they are able to form functional synapses [26, 28]. To analyze whether editing the *flailer* gene rescues the transport of synaptic proteins and does not show a broad distribution along dendrites of the cargo, we performed immunofluorescence staining using antibodies against Synaptophysin (pre-synaptic protein) and PSD95 (post-synaptic protein). We found that, similar to wild-type neurons, FL1-treated FL neurons display defined clusters of PSD95 and Synaptophysin, compared to Flailer neurons, where the expression of PSD95 is distributed broadly throughout the dendritic shafts and opposing to Synaptophysin signal after 10 days of infection (Fig. 1F). Taken together, our data demonstrate that we can edit the *flailer* genomic locus, resulting in a reduction of its expression that can rescue the synaptic transport defect in FL-cultured neurons.

### Synaptic transmission defects in FL neurons are recovered after *flailer* gene editing

**Fig. 2:**
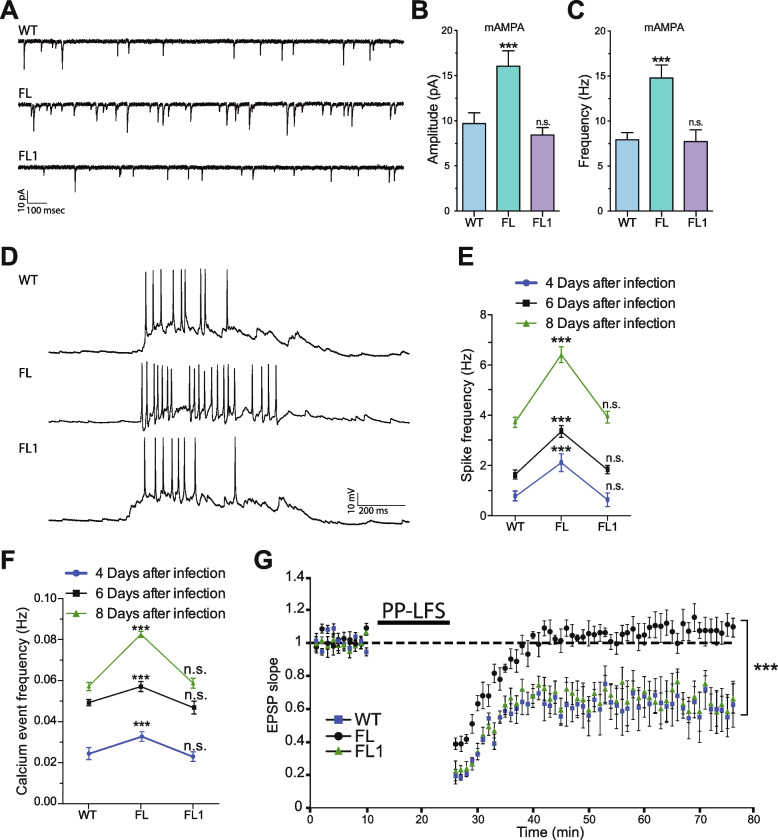
Synaptic transmission defects are corrected by *flailer* gene editing. **A** Representative traces of miniature AMPA receptor currents from wild-type, FL, and FL1 infected neurons. Quantification of the amplitude (**B**) and frequency (**C**) of currents. **D** Representative traces of current clamp recordings showing spiking of neurons. **E** Spike frequency of wild-type, FL, and FL1 neurons at 4, 6, or 8 days after infection. For **A**–**E** At least 20 neurons for each condition from 3 independent neuronal cultures were analyzed. **F** Calcium imaging using GCamp6 at 4–6–8 days after infection. A total of 500 neurons from at least 3 independent neuronal cultures were analyzed. **G** LTD induction by PP-LFS in hippocampal slices of wild-type, FL, and FL1 animals at P22-23 after HSV infection. 9 slices from 3 different animals were used for each condition. Bars represent mean ± SEM; ***p < 0.001. One-way ANOVA was used to determine significance compared to wild-type condition

Due to the defects in synaptic transport and the lack of LTD caused by the dominant negative nature of the FL protein, FL neurons exhibit an increased number of synapses localized in the dendritic shafts, resulting in enhanced synaptic transmission [26, 28]. To determine whether editing the *flailer* gene can recover synaptic transmission defects, we conducted patch-clamp recordings and calcium imaging of wild-type, FL, and FL1 cultured neurons. Patch-clamp recordings indicate that the amplitude and frequency of mAMPA receptor currents, which are enhanced in FL neurons, decrease in FL1 neurons comparable to wild-type cells (Fig. 2A–C). Furthermore, current-clamp recording used to measure the spiking of neurons revealed that FL1-treated neurons exhibit spike frequencies similar to wild-type neurons (Fig. 2D–E). As expected, we observe an increase in spiking frequency with development from 4–8 days after infection (Fig. 2E). Interestingly, spike frequency was restored to wild-type levels as early as 4 days after infection was conducted to correct the mutation (Fig. 2E). This finding is supported by calcium imaging using the genetically encoded probe GCaMP6, which reveals that FL1-treated neurons have calcium event frequencies that are similar to wild-type neurons and reduced frequencies compared to FL neurons as early as 4 days after infection (Fig. 2F). Similar to current clamp recordings, calcium imaging demonstrates that synaptic activity in neurons increases during the developmental period of 4–8 days after infection (Fig. 2F).

Previously we demonstrated that FL animals exhibit normal long-term potentiation (LTP), but LTD by paired-pulse low-frequency stimulation (PP-LFS) is absent both in the visual cortex [26] and hippocampus [28]. To further investigate this phenomenon, we employed HSV viruses to express mRuby3 alone or in combination with FL1 into the hippocampus at postnatal day 20 (P20). After 48–72 h, acute hippocampal slices from wild-type, FL, and FL1 animals were prepared and recorded. Consistent with previous reports, FL slices were unable to induce LTD, whereas FL1 and wild-type slices exhibited robust LTD induction by PP-LFS (Fig. 2G). Taken together, our data demonstrate that the editing of the *flailer* gene using FL1 can restore synaptic function both in vitro and in vivo, reaching levels comparable to those of wild-type neurons.

### Targeted DN-CRISPR editing of the *flailer* gene in the ventral hippocampus ameliorates some of the behavioral phenotypes in FL animals in vivo

**Fig. 3:**
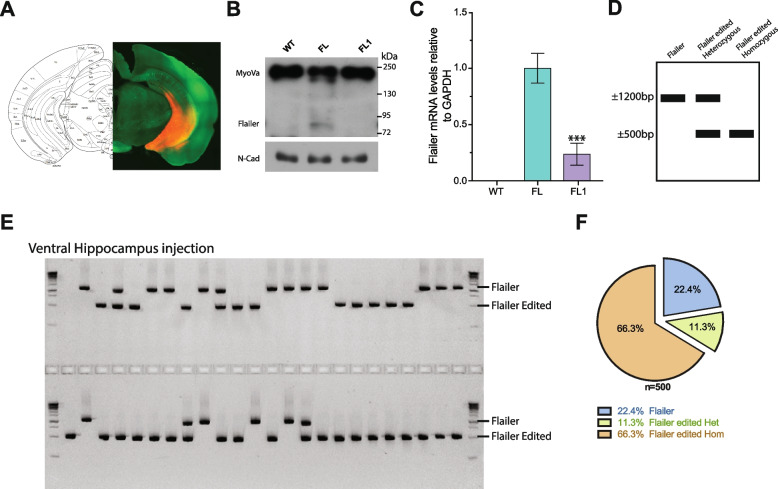
Injection of FL1 AAV into the ventral hippocampus results in highly efficient *flailer* gene editing. **A** Representative image of ventral hippocampus stereotactic injection showing a high infection rate by TdTomato signal at the targeted region. Infected ventral hippocampus tissue was extracted to determine Protein (**B**) and mRNA (**C**) expression levels from wild-type, FL, and FL1-injected animals. mRNA levels are relative to FL animals and GAPDH was used to normalize. **D**–**F** Single nuclei PCR to determine gene editing in FL1icv injected Flailer animals. **D** Scheme depicting the expected PCR products of FL animals (~ 1200 bp), FL edited heterozygous (~ 1200 bp +  ~ 500 bp), and FL edited homozygous (~ 500 bp). **E** Representative gel of PCR products from single nuclei FL1 ventral hippocampus infected tissue. **F** Quantification of the number of FL, FL heterozygous, and FL homozygous products. A total number of 500 cells were analyzed from 10 FL1 ventral hippocampus-injected animals

We have previously characterized the behavioral phenotype of Flailer animals, demonstrating early onset epilepsy, memory deficits, repetitive behaviors, and increased anxiety [28]. Building upon our in vitro and in vivo findings that FL1 corrects synaptic transport and transmission, we aimed to investigate whether delivering FL1 into a specific brain region could mitigate some of the behavioral phenotypes associated with the flailer mutation. We focused on the ventral hippocampus for gene editing, as this region has been widely implicated in anxiety and memory formation [30, 31]. Bilateral delivery of Tdtomato alone or in conjunction with FL1 was achieved via stereotactic injections at P24–P26 (Fig. 3A). Four weeks later, animals underwent behavioral testing before being sacrificed to determine the accuracy of injection location, gene editing efficiency, and changes in Flailer expression.

To assess alterations in Flailer expression, we isolated infected ventral hippocampal tissue from wild-type, Fl, and FL1-injected animals. Notably, FL1-injected animals showed a 70% reduction in Flailer protein (Fig. 3B) and mRNA levels (Fig. 3C). To evaluate editing efficiency, DNA was extracted from infected ventral hippocampal tissue and subjected to single nuclei PCRs analysis. In this assay, a ~ 1200-bp band indicates the presence of a wild-type *flailer* gene, whereas a ~ 500-bp band suggests successful genomic locus editing (Fig. 3D). Single nuclei PCR analysis was performed on 500 individual nuclei from 10 FL1-injected animals. Results revealed that 66.3% of the analyzed cells had both copies of the *flailer* gene edited, 11.3% of neurons exhibited editing in only one copy, and 22.4% showed no editing at all (Fig. 3E–F), demonstrating high gene editing efficiency in vivo. To be included in the behavioral analysis, animals had to display clear ventral hippocampal expression of fluorescent protein and exhibit a minimum reduction of 60% in Flailer mRNA expression.

**Fig. 4:**
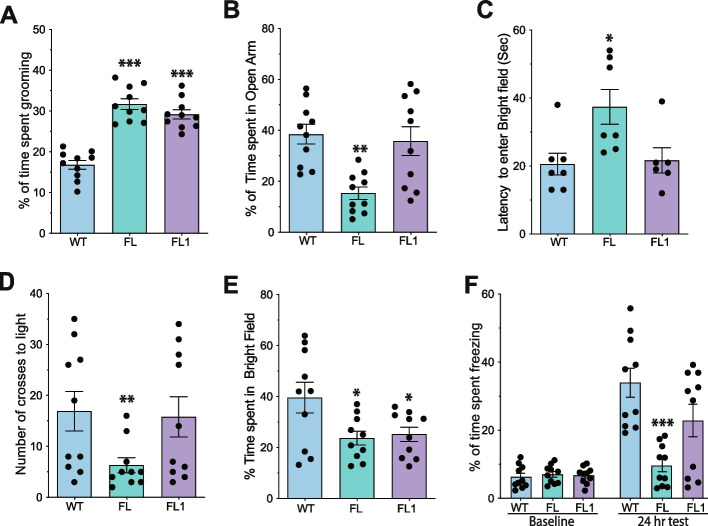
FL1 AAV injection into the ventral hippocampus restores memory formation and partly anxiety behaviors of FL animals. **A** Percentage of time in a 1-h session that animals spend grooming. **B** Elevated plus maze test to determine the percentage of time spent in the open arms. **C**–**E** Light and dark test measuring the latency to enter the bright field (**C**), number of crosses to light (**D**), and percentage of time in bright field (**E**). **F** Contextual fear conditioning test to measure long-term memory formation (24 h). *N* = 10 animals per condition were analyzed. Bars represents mean ± SEM; *p < 0.05, **p < 0.01, ***p < 0.001. One-way ANOVA was used to determine significance compared to wild-type condition

To investigate the recovery of behaviors associated to ASD, we initially focused on grooming behavior. As previously reported, FL animals spend approximately 30% of their time engaged in grooming [28], a significantly higher percentage than wild-type animals. Interestingly, animals injected with FL1 in the ventral hippocampus exhibited a similar grooming behavior percentage as observed in FL mice, indicating that this behavior was not reversed in FL1-injected animals (Fig. 4A). To assess anxiety behaviors, we utilized the elevated plus-maze and light and dark tests. The elevated plus-maze test measures the time an animal spends in the open or closed arms. Animals exhibiting anxiety tend to spend more time in the closed arms. Our findings demonstrate that FL1-injected animals spent more time in the open arms compared to FL animals, reaching levels comparable to wild-type animals (Fig. 4B). This suggests that FL1-injected animals exhibit reduced anxiety compared to their FL littermates. The light and dark test consists of two chambers one dark, and one highly illuminated connected by a door. Anxious animals tend to stay in the dark side and avoid exploring the illuminated side. By measuring these parameters, we can estimate anxiety behavior. Our findings reveal that FL1-injected animals entered the light side faster and more frequently than FL animals, reaching levels comparable to those observed in wild-type animals (Fig. 4C–D). Interestingly, FL1 animals did not remain in the brightly illuminated area for as long as wild-type animals (Fig. 4E), suggesting that eliminating the FL mutation in the ventral hippocampus alone is insufficient for the complete recovery of the anxiety behaviors exhibited by Flailer animals. Next, we evaluated long-term memory formation (24 h) using the contextual fear conditioning paradigm. We have previously demonstrated that FL animals struggle to form long-term memory in the contextual fear conditioning apparatus, evident through a significant reduction in freezing behavior [28]. Encouragingly, our findings indicate that similar to wild-type animals, FL1-injected animals spent significantly more time freezing than FL animals (Fig. 4F), the latter of which do not demonstrate strong memory formation as previously described [28]. Collectively, our results demonstrate that editing the *flailer* gene in the ventral hippocampus can significantly enhance memory formation and partially alleviate anxiety levels in Flailer animals.

### Intracerebroventricular injection of DN-CRISPRs fully recovers behaviors in FL animals

**Fig. 5:**
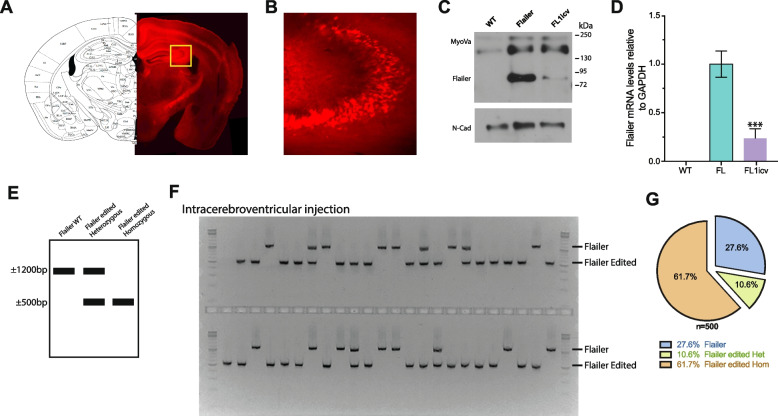
FL1icv AAV injection results in highly efficient *flailer* gene editing. **A** Representative image of intracerebroventricular injection showing high and extensively distributed infection by TdTomato signal. **B** Magnification of the hippocampus area is depicted in a yellow square in **A**. Protein (**C**) and mRNA (**D**) expression levels of infected tissue extracted from the cortex of wild-type, FL, and FL1icv-injected animals. **E** Scheme depicting the expected PCR products of FL animals (~ 1200 bp), FL edited heterozygous (~ 1200 bp +  ~ 500 bp), and FL edited homozygous (~ 500 bp). **F** Representative gel of PCR products from single nuclei FL1icvl infected cortical tissue. **G** Quantification of the number of FL, FL heterozygous, and FL homozygous products. A total number of 500 cells were analyzed from 10 FL1icv animals. Bars represent mean ± SEM; ***p<0.001. One-way ANOVE was used to determine significance compared to FL condition

Given that injection of FL1 in the ventral hippocampus of FL animals improved memory formation and alleviated certain anxiety behaviors, we investigated whether whole brain editing of the *flailer* gene through intracerebroventricular injection could restore entirely the characteristic behaviors observed in Flailer animals [28]. For this, we delivered the CRISPR/Cas9 system through AAVs encapsulated with the PHP.eB capsid [32]. This approach allows the broad and efficient infection of brain tissue, as we have previously reported to knock out KMT2C and induce ASD-like phenotypes in mice [13]. In this context, we packaged tdTomato alone or co-delivered it with FL1 and Cas9n (henceforth FL1icv) into AAVs employing the PHP.eB capsid, and then proceeded to administer them intracerebroventricularly to wild-type and FL animals at P0. Eight weeks after injections, a series of behavioral tests were conducted to evaluate anxiety and memory formation in the animals. Following the completion of these tests, the animals were subsequently sacrificed, allowing for an assessment of infection efficiency, Flailer expression, and gene editing efficiency. As anticipated, the use of AAVs with the PHP.eB capsid for infection led to a robust and widespread infection, evident from the TdTomato signal, with a significant concentration in the hippocampus and cortex, as well as a broad distribution throughout the animal’s brains (Fig. 5A–B). Using infected cortical tissue, we quantified the expression levels of Flailer post-editing. FLicv animals exhibited a significant ~ 70% reduction in Flailer protein expression (Fig. 5C) and mRNA levels (Fig. 5D), when compared to their FL counterparts. To analyze the editing efficiency reached within the infected cortical tissue, we conducted single nuclei PCR on 500 nuclei derived from FL1icv injected animals. This analysis revealed that among the total cells analyzed, 61.7% displayed editing in both copies of the *flailer* gene*,* 10.6% exhibited editing in a single copy, and 27.6% remained unedited (Fig. 5F–G). Animals exhibiting high and broad infection, coupled with a minimum reduction of 60% in Flailer mRNA expression, were considered for the subsequent behavioral analysis.

**Fig. 6:**
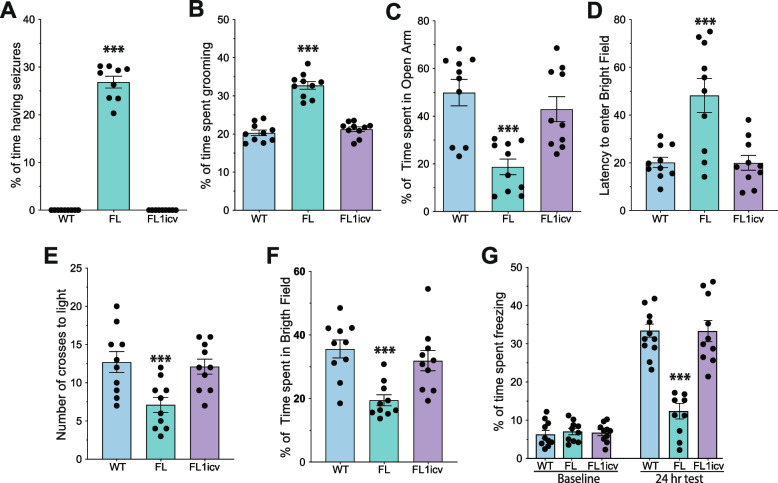
Intracerebroventricular injection of FL1 recovers ASD and anxiety-like behaviors of FL mice. **A** Quantification of the time animals spend having seizures. **B** Percentage of time in a 1h session that animals spend grooming. **C** Elevated plus maze test to determine the percentage of time spent in the open arms. **D**–**F** Light and dark test measuring the latency to enter the bright field (**D**), number of crosses to light (**E**), and percentage of time in bright field (**F**). **G** Contextual fear conditioning test to measure long-term memory formation at 24 h. *N* = 10 animals per condition were analyzed. Bars represent mean ± SEM; ***p < 0.001. One-way ANOVA was used to determine significance compared to wild-type condition

Flailer animals are recognized for their severe seizure episodes, typically occurring until around P30, after which these episodes abruptly cease [28]. To determine the percentage of time during which the animals experience seizures, we conducted a 1 h long session recording and manually calculated the time the animals spent seizing. We observed that FL1icv animals in the range of P20–P25 showed no signs of seizures, in contrast to Flailer littermates who manifested seizures for roughly 25% of the analyzed time span (Fig. 6A, Additional file 2: video 1). Next, we analyzed grooming behavior by putting the animals in a cage for 1 h and recording their behavior. As we have previously shown, FL animals spend 35% of their time grooming [28], in contrast, both wild-type and FL1v animals exhibited a grooming duration of approximately 15%, indicating a reversal of the grooming condition (Fig. 6B). To analyze the presence of anxiety-related behaviors, we employed both the elevated plus maze and light and dark tests. We have previously shown that FL animals have increased anxiety compared to wild-type animals [28]. In the elevated plus maze test, our findings revealed that FL1icv animals spent more time in the open arm compared to FL littermates, aligning their behavior with that of wild-type animals (Fig. 6C). Utilizing the light and dark test, we observed that FL1icv animals exhibit a reduced latency to enter the bright field in comparison to FL animals, akin to the behavior observed in wild-type animals (Fig. 6D). Furthermore, similar to wild-type animals, FL1icv animals demonstrated a significant increase in the number of crossings (Fig. 6E) and the percentage of time spent in the light compartment (Fig. 6F), as opposed to their FL littermates. Lastly, we tested the animals for long-term memory formation at 24 h using the contextual fear conditioning paradigm. Our results indicated that FL animals showed reduced freezing times compared to wild-type animals, while both wild-type and FL1icv animals displayed comparable levels of freezing behavior (Fig. 6G). Our findings demonstrate that intracerebroventricular injection of AAVs carrying the FL1 gene editing construct effectively and efficiently reinstates the distinctive behaviors associated with Flailer animals. This recovery encompasses the reduction of seizures, amelioration of repetitive behaviors, reduction of memory deficits, as well as mitigation of anxiety behaviors.

## Discussion

Numerous neurodegenerative diseases, including ASD and Alzheimer’s disease, have been strongly linked to genomic rearrangements, including gene duplications that result in abnormal gene dosage [1, 2, 33–35]. Despite the successful application of the CRISPR/Cas9 technology targeting diverse genes in mice to excise specific genomic sequences [36–39], developing gene therapy approaches to address genomic duplications has proven challenging, demanding a high level of precision to evade interference with the native copies of the duplicated genomic sequence. Moreover, some duplications have structures where extensive DNA fragments need to be removed to achieve gene expression silencing. In this study, we harnessed the CRISPR/Cas9 system, specifically DN-CRISPRs [14–17], to precisely target a partial genomic duplication that produces ASD and anxiety-like behaviors in mice. One limitation of using DN-CRISPRs for gene editing is the requirement for close proximity of sgRNAs (offset). Previous studies have shown that when sgRNAs are separated by more than 100 bp [14] or, at most, 300 bp [17] excision efficiency diminishes significantly. Here we demonstrate the feasibility of using DN-CRISPR to target and remove a larger fragment (> 700 bp) as part of a partial genomic duplication. To our knowledge, this is the first demonstration that a longer stretch of DNA can be efficiently removed by DN-CRISPRs. Thus, this approach holds the potential to target the precise removal of longer DNA fragments with higher specificity compared to the double-stranded breaks induced by wild-type SpCas9. In contrast to previous assumptions, our findings suggest that using DN-CRISPR with sgRNAs featuring large offsets is indeed feasible and can be equally or even more efficient than using sgRNAs with small offsets. It is important to note that this insight should be taken into account when considering off-target effects of DN-CRISPRs. During our experimentation, a significant number of sgRNAs were assessed. Despite this extensive testing, no common feature leading to effective gene editing with large offsets was discovered. Consequently, the determination of the optimal sgRNA pair for each gene requires a trial-and-error approach.

We targeted the Flailer partial genomic duplication resulting in a reduction of Flailer expression in vitro and in vivo*.* Significantly, we underscore the specificity of our methodology as endogenous copies of *gnb5* and *moy5a*, elements partially targeted by FL1 DN-CRISPRs, remained unaltered with no accompanying changes in their expression. Our findings represent the pioneering instance wherein extending the offset between sgRNAs within DN-CRISPRs has facilitated the precise targeting and elimination of genomic regions. This expansion broadens the applicability of gene editing strategies for addressing genomic duplications associated with neurodegenerative diseases like ASD, wherein gene duplications, involving genes such as SHANK3, NRXN1, or NLGN1, have emerged as contributory factors [1–6]. Our approach holds the potential to extend to other genetic disorders characterized by genomic duplications, thereby introducing a novel avenue for the advancement of gene therapies. We do acknowledge that the Flailer mutation is unique in terms of its genomic rearrangement, and as a result, developing strategies for targeting other genomic duplications might indeed be more complex.

The Flailer animal was first described by Jones et al. (2000) [18] showing its role as a dominant negative of MyoVa. In wild-type neurons MyoVa moves to the positive end of actin filaments [22], transporting key components to the synapse such as receptors, scaffolding proteins, and mRNAs [24–27]. The Flailer mutation impairs the ability of MyoVa to transport these components, resulting in decreased numbers of synaptic spines, increased AMPA receptor-mediated currents, and the lack of LTD [26]. Our experiments in vitro demonstrated that upon editing of the Flailer gene, the transport of PSD95, a key scaffolding protein within the synapse, was recovered exhibiting PSD95 clusters similar to those in wild-type neurons.

Our experiments have unveiled that the Flailer animal model exhibits increased synaptic activity and reduced LTD [26, 28], a phenomenon consistent with other ASD models [40–42]. This observation implies that the failure of LTD to properly eliminate synapses may contribute to this phenotype. By precise targeting of the *flailer* gene using DN-CRISPRs, we were able to restore LTD induction in hippocampal slices in vivo and a concurrent restoration of spontaneous activity and spiking of neurons was observed. The impairment of LTD not only in the Flailer model but also in other mutant mouse models such as TSC1, Pcdh10, or Pten has been consistently linked to the development of ASD [28, 40, 41, 43]. The dysfunction in LTD and the resulting hyperconnectivity between neurons leads to an absence of activity-dependent synapse elimination, often referred to as “pruning,” during the developmental phase. The noticeable lack of LTD implies that the process of LTD and the associated synapse elimination are notably vulnerable to disruption during brain development.

The use of whole-genome knockouts or conditional knockouts with specific promoters has been a common approach to investigate the role of specific brain regions or cell types in governing synaptic transmission and behavior. Having the whole brain intact and selectively disrupting the targeted area allows to test the necessity of a particular brain area within a given neural circuitry for orchestrating a specific behavior. Conversely, the Flailer animal model presents a unique opportunity to study the sufficiency of brain regions in governing specific behaviors. The broad expression of the Flailer protein in the brain causes a dominant negative effect on MyoVa, resulting in the global disruption of brain function. By leveraging region-specific AAV delivery of DN-CRISPR-mediated gene editing, we can meticulously restore specific brain areas and investigate whether these regions alone are sufficient to modulate specific behaviors, as demonstrated for the ventral hippocampus. Through this approach, we can use the Flailer model to pinpoint the neural circuits and brain regions implicated in behaviors related to human neurological diseases.

Flailer animals exhibit a range of behavioral phenotypes, including early-onset epilepsy, anxiety, repetitive behaviors, and memory deficits [28], which resemble those seen in ASD and anxiety-like animal models [44]. Our initial focus centered on the ventral hippocampus, a brain region that has been implicated in the regulation of anxiety and memory formation [26, 27]. Our experiments targeting the ventral hippocampus showed that memory formation is recovered while the restoration of anxiety behavior remained only partial. This implies that anxiety behaviors are influenced to some extent by the ventral hippocampus, yet editing of this region alone is not sufficient to fully recover these behaviors through gene editing of the Flailer mutation.

To evaluate the possibility to fully recover the animal phenotype, we turned to intracerebroventricular delivery of FL1 using the high infectivity PHP.eB capsid [32], as we have previously done [13]. This capsid has been previously used to deliver CRISPR/Cas9 to produce the knockout of KMT2C and determine its association to ASD [13], and in an Alzheimer’s model, resulting in improvement in cognitive performance and reductions in Alzheimer’s markers such as amyloid-beta deposition [36], among others. Our results unequivocally demonstrate that post-brain injection of FL1 in Flailer animals, we achieved a complete restitution of all assessed behaviors, encompassing epilepsy, grooming behavior, anxiety, and memory formation. The attainment of full recovery can be attributed to the remarkably high genome editing efficiency in vivo, with approximately 70% of at least one copy of *flailer* undergoing editing, while endogenous copies of *gnb5* and *myo5a* remained unaltered. This degree of editing markedly surpasses other studies where DN-CRISPRs [14] or CRISPRs [36] were employed for gene editing, typically achieving efficiencies of approximately 30%. These results demonstrate that gene therapy in mice using DN-CRISPRs can efficiently edit the genome, resulting in the recovery of synaptic and behavioral phenotypes in the animals. Remarkably, our findings showcase the attainment of reversion across a wide array of phenotypes, encompassing seizures, grooming behaviors, and cognitive tests. This underscores the promising potential of utilizing DN-CRISPR as a viable avenue for the development of neurological disease therapies.

Altogether, our findings demonstrate that the utilization of DN-CRISPRs represents an efficacious and proficient approach for precisely targeting genomic duplications, thereby opening new opportunities for therapeutic interventions where genomic aberrations need to be amended.

## Conclusions

We extend the application of DN-CRISPRs by demonstrating their efficacy in excising a genomic region that is greater than > 700 bp long. This not only expands the horizons of gene editing techniques but also capitalizes on the augmented precision that DN-CRISPR offers. Leveraging AAV viral delivery, we exhibit remarkable gene editing efficiency (> 60%) in eliminating the Flailer partial genomic duplication. The substantial editing levels enable the restoration of ASD/anxiety-related phenotypes in Flailer mice. Collectively, our findings underscore the potential of DN-CRISPR as an innovative therapeutic avenue for rectifying genomic aberrations.

## Methods

### Animals

All protocols involving rodents were carried out according to NIH and ARRIVE guidelines. Protocols were approved by the MIT Committee on Animal Care (CAC) and the Ethical and Bio-security Committees of Universidad Andres Bello. Flailer (Jackson Laboratories Strain #:000502) and wild-type (Jackson Laboratories Strain #:000664) animals were used for behavioral and in vitro analyses.

### DNA constructs

To express SpCas9n (Streptococcus pyogenes Cas9), the coding sequence was obtained from pX335 (Addgene #42,335) and subcloned into a AAV backbone under the MeCP2 promoter as in [45]. 20nt target sequences were selected contiguous to a 5′-NGG photospacer-adjacent motif (PAM) sequence in both sense and antisense strands. sgRNA were targeted to the myo5a mixed intron (intron 3) or the second gnb5 exon in the *flailer* gene (Fig. 1A). Each pair of sgRNAs (1 sense/1 antisense strand) were synthesized by gBlocks (IDT, USA) under the human U6 promoter and subcloned into an AAV backbone vector coding for tdTomato or Azurite under the hSyn1 promoter. Both sgRNAs were delivered in the same vector. sgRNA pair sequences were designed with the first sgRNA targeting exon 2 of *gnb5* and the second targeting the intron part of *myo5a* for specificity. Sequences are as follows: FL1 (*gnb5*: 5′ CTTTGCACCAATCCATGCAC 3′; *myo5a:* 5′ ATGTTCATGCTTCTATTGAC 3′); FL2 (*gnb5*: 5′ ACAAAGTCCTGTGCATGGAT 3′; *myo5a:* 5′ ATAAAAATTAGTACATGTAT 3′); FL3 (*gnb5*: 5′ TGCACAGGACTTTGTTCCCG 3′; *myo5a:* 5′ ATGTTCATGCTTCTATTGAC 3′); FL4 (*gnb5*: 5′ ACAAAGTCCTGTGCATGGAT 3′; *myo5a:* 5′ CTTACCACGTATAAGATGCT 3′); FL5 (*gnb5*: 5′ CTTTGCACCAATCCATGCAC 3′; *myo5a:* 5′ TGAACCAAGCATCTTATACG 3′). For calcium imaging, we used a plasmid coding for GCamP6s under the CamKIIa promoter in a AAV backbone. AAV-CaMKIIa-GCaMP6s-P2A-nls-dTomato was a gift from Jonathan Ting (Addgene plasmid # 51,086; http://n2t.net/addgene:51086; RRID:Addgene_51086).

### Cell line cultures and transfections

Neuro-2a (N2a) and HEK293T cells were grown in DMEM containing 10% Fetal bovine serum (FBS). Cells were maintained at 37 °C in 5% CO_2_ atmosphere. Cells were transfected using Lipofectamine 3000 or Polyethylenimine (PEI) “MAX” reagent (Polysciences, Cat 24,765), according to manufacturer’s protocols.

### Primary neuron cultures

Postnatal day 0 (P0) Flailer or wild-type animals were euthanized by decapitation and the whole brain was extracted in ice-cold Ca^2+^/Mg^2+^-free Hank’s balanced salt solution (HBSS) solution. Meninges were removed, and the tissue was minced and incubated with Papain (20 units) for 15 min at 37 °C. Cells were rinsed twice with HBSS, resuspended by mechanical agitation through fire-polished glass Pasteur pipettes of decreasing diameters, and plated over poly-L-lysine-coated culture plates or coverslips. Cultures were maintained at 37 °C in 5% CO_2_ in growth media (Neurobasal-A (Life Technologies 1,088,802) supplemented with B27 (Life Technologies 17,504,044), 2 mM L-glutamine (Life Technologies 25,030–081), 100 U/ml penicillin/streptomycin (Life Technologies 15,070–063)]. Half of the media was replaced every 3 days.

### AAV production

For infection of cultured neurons, low titer viral particles were produced using AAV1/AAV2 or PHP.eB [32] capsids. Briefly, HEK293T cells were grown to approximately × 10^4^ cells/cm^2^ with DMEM 10% FBS. Cultures were transfected using PEI “MAX” reagent (Polysciences, Cat 24,765) with plasmids coding for the capsids, the transfer vectors (sgRNAs with fluorophores, SpCas9n, or GCamP6), and the helper plasmid pDF6. 24 h after transfection, the media was exchanged for DMEM 1% FBS. After 72 h, media and cells were collected from the plates, centrifuged and the cell pellet was lysed by freeze–thaw cycles. The supernatant was filtered with 0.45μm syringe filters stored at − 80 °C for posterior use. High-titer viral particles for injections were prepared as in [13, 46]. Briefly, HEK 293 T cells were grown to approximately 6 × 10^4^ cells/cm2 with DMEM 10% FBS. Cultures were transfected using PEI “MAX” reagent (Polysciences, Cat 24,765) with PHP.eB capsid plasmids, the transfer vectors (sgRNAs with fluorophores, or SpCas9n), and the helper plasmid DF6. After 24 h of transfection, the media was exchanged for DMEM 1% FBS. 72 h later, media was collected from the plates and replaced with fresh DMEM 1% FBS. The collected media was stored at 4 °C. 120 h after transfection, the cells were detached from the plate and transferred to 250 mL conical tubes, together with the collected media. Cells were centrifuged for 10 min at 2000 g, and the supernatant was removed and saved for later use. The pellet was resuspended in SAN digestion buffer (5 mL of 40 mM Tris, 500 mM NaCl, and 2 mM MgCl_2_ pH 8.0) containing 100 U/mL of Salt Active Nuclease (Arcticzymes, USA) and incubated at 37 °C for 1 h. The supernatant was precipitated using 8% PEG 8000 and 500 mM NaCl. It was incubated on ice for 2 h and centrifuged at 4000 g for 30 min in 250 mL bottles. The supernatant was collected and resuspended with SAN digestion buffer. The solution was placed in an iodixanol gradient and ultracentrifuged at 350,000 g for 2.5 h. The phase containing the AAV was rescued and frozen at − 80 °C for later use.

### Immunofluorescent staining

Immunocytochemistry was performed as described [26, 47, 48]. Briefly, 10–12 DIV neurons were fixed with 4% paraformaldehyde (PFA) and permeabilized with 0.1% Triton X-100 for 5 min. Cells were blocked with 1% BSA for 30 min at 37 °C and incubated with primary antibodies overnight at 4 °C. Primary antibodies used were: PSD95 (Neuromab 75–028), Synaptophysin (Zymed 18–0130), and mCherry (Biorbyt orb153320). Cells were rinsed three times with PBS and incubated for 2–3 h at room temperature with appropriate secondary antibodies conjugated to Alexa-488, Alexa-546, or Alexa-647 (Life Technologies). Cells were coverslipped using Prolong Gold and imaged on a Nikon C2 + confocal microscope with a × oil immersion objective (Nikon Instruments Inc., Melville, NY, USA).

### Purification of cell nuclei and sorting

Animals injected with AAV coding for FL1 were sacrificed and infected brain tissue was quickly extracted in ice-cold PBS using a fluorescent dissecting scope. Samples were directly used, or shock-frozen on liquid nitrogen. Nuclei purification was performed as described in [45]. Briefly, dissected tissue was homogenized in 2 ml ice-cold homogenization buffer (HB) (320 mM sucrose, 5 mM CaCl, 3 mM Mg(Ac)_2_, 10 mM Tris pH7.8, 0.1 mM EDTA, 0.1% NP40, 0.1 mM PMSF, 1 mM β-mercaptoethanol) using 2 ml Dounce homogenize; 25 times with pestle A, followed by 25 times with pestle B. HB was added to complete 5 ml and kept on ice for 5 min. Five milliliters of 50% OptiPrep density gradient medium containing 5 mM CaCl, 3 mM Mg(Ac)_2_, 10 mM Tris pH 7.8, 0.1 mM PMSF, and 1 mM β-mercaptoethanol was added and mixed. The resulting lysate was loaded on top of 10 ml 29% iso-osmolar OptiPrep solution in a conical 30 ml centrifuge tube (Beckman Coulter, SW28 rotor). Samples were centrifuged at 10,100 × g (7,500 r.p.m.) for 30 min at 4 °C. The supernatant was removed, and the nuclei pellet was gently resuspended in 65 mM β-glycerophosphate (pH 7.0), 2 mM MgCl_2_, 25 mM KCl, 340 mM sucrose, and 5% glycerol. Extracts were controlled for purity using bright field microscopy. Purified intact nuclei were sorted into single-cell nuclei using BD FACSAria III (Koch Institute Flow Cytometry Core, MIT). Nuclei were sorted in 5 μl of QuickExtract DNA Extraction Kit (Epicentre, USA) in 96-well format.

### Single nuclei PCR

Extracted DNA from sorted single nuclei from Flr and Flr-FL1 neurons was used for PCR analyses to determine the editing of alleles in single nuclei. PCR was conducted using nested PCR primers with a first amplification step of 40 cycles using primers: 5′ AAGAAAGCTTTGCATGCCCT 3′, 5′ AATGGCTCCAAACCAACACA 3′; followed by 25 cycles with a second set of primers: 5′ AGCTTTGCATGCCCTAAGAA 3′, 5′ CTCCAGGGCAATTGTAGCCA 3′. Samples were run in agarose gels to visualize allele editing.

### Genomic DNA extraction and editing efficiency

Primary cortical neurons were infected at 3 DIV with FL1 and at 10–12 DIV neurons were harvested and DNA extracted using Quick-DNA Miniprep Kit (Zymo Research, USA) following manufacturer recommendations. For tissue, injected animals with AAV coding for FL1 were sacrificed and infected brain tissue was quickly extracted in ice-cold PBS using a fluorescent dissecting scope. Tissue was digested with Proteinase K and purified using a Quick-DNA Miniprep Kit (Zymo Research, USA) following the manufacturer’s protocol. To test Flailer editing, PCR with primers encompassing the edited region (5′ AGCTTTGCATGCCCTAAGAA 3′, 5′ CTCCAGGGCAATTGTAGCCA 3′) were used to determine editing of the locus. PCR products were run in agarose gels and purified by a Gel extraction kit (Qiagen). DNA was purified cloned into a TOPO-TA (Life Technologies, USA), and sent for Sanger sequencing to corroborate editing of the gene. To test specificity of sgRNA against Flailer, SURVEYOR assay against *gnb5* or *myo5a* was performed using SURVEYOR nuclease assay (IDT, USA) using the following primers: *gnb5* sense 5′ AATTGTGGTCCTGTCCTTGC 3′, antisense 5′ TGGGTTCCTTCACAAATCCT 3′, myo5a sense 5′ TTACAATGAAGACGCTGTGGA 3′, antisense 5′ CTTTACTGTCCCCACCATGG 3′.

### Electrophysiology

Whole-cell patch-clamp recordings were performed on Flr and wild-type-Flr primary cortical neurons as previously described [26, 49, 50]. Briefly, neurons were transferred to an external solution containing (in mM) 150 NaCl, 5.4 KCl, 2.0 CaCl2, 2.0 MgCl2, 10 HEPES (pH 7.4), and 10 glucose. Patch electrodes (7–10 MΩ) were filled with (in mM) 120 CsCl, 10 BAPTA, 10 HEPES (pH 7.4), 4 MgCl2, and 2 ATP-Na2. After high resistance seal and break in (> 1GΩ), whole cell voltage or current was recorded using an Axopatch 700B amplifier (Molecular Devices). Signals were low pass filtered (5 kHz) and digitized (5–40 kHz) using PClamp 10 software. For voltage-clamp recordings, neurons were held at − 60 mV. To record miniature AMPA excitatory post-synaptic currents (EPSC); AP5, strychnine, bicuculline, and TTX were added to the external solution.

For LTD experiments, acute slices were prepared from the hippocampus of wild-type, Flr, or Flr-injected animals, as previously described [26, 28]. Injections were performed at P17–P18 with HSV virus coding for Cas9n and FL1 (sgRNAs). Recordings were performed 2–3 days later. Animals were anesthetized with isoflurane and decapitated. Brains were quickly removed and chilled in an ice-cold dissection buffer. Transverse dorsal hippocampal 350–400 micron sections were cut using a VT-1000S vibratome (Leica, Germany) in ice-cold carbogenated sucrose solution containing (in mM) 240 sucrose, 2.5 KCl, 1 CaCl_2_, 5 MgSO_4_, 26 Na_2_HCO_3_, 1NaH_2_PO_3_, 11 glucose, transferred to a chamber containing carbogenated artificial cerebrospinal fluid (ACSF) for 30 min at 32 °C then maintained at RT (22–24 °C) for a minimum of 1 h prior to recordings. Electrodes were pulled to 3-5MΩ-tip resistance using a P-97 puller (Sutter Instruments, CA). Stimulating electrodes were fabricated tungsten bipolar electrodes (WPI) driven by ISO-STIM-01D (NPI Electronic Gmb). For recordings, slices were submerged and perfused (3 ml/min) in a carbogen-saturated ACSF at room temperature. Neurons positive for mRuby2 were selected for recording in the stratum radiatum of CA1. LTD was induced by paired-pulse low-frequency stimulation (PP-LFS) consisting of 900 paired pulses delivered at 1 Hz. Signals were amplified with a MultiClamp 700B, digitized with a Digidata 1440A, filtered at 2 kHz, sampled at 10 kHz, and analyzed using pClamp 10 (Mol. Devices Corp., CA).

### Calcium events

Wild-type and Flailer primary neuronal cultures were infected at 3 DIV with AAV coding for GCaMP6s (Addgene #51,086) and FL1 or control AAVs. 4–8 days after infection cells were placed into a temperature and CO_2_-controlled chamber (Tokai-Hit) over a Nikon TE-2000 epifluorescent microscope with a LED illumination system and a high-speed Zyla camera (Andor, Ireland). Images were acquired every 30 ms for 10 min. Regions of interest were selected, and changes of fluorescence overtime calculated. Fluorescence peaks in 10 min were counted to calculate the frequency of calcium events.

### Western blot analysis

Whole homogenate fractions were prepared from infected cultured neurons or brain tissue from wild-type, FL or FL1 cells. For tissue, injected animals with AAV coding were sacrificed and infected brain tissue was quickly extracted in ice-cold PBS using a fluorescent dissecting scope. 10 mg of tissue was homogenized with a dounce and incubated for 30 min in 500μl of RIPA buffer on ice. Extracts were centrifuged for 15 min at 4°C and supernatant was used for analyses. Protein concentrations were determined using BCA (Pierce, IL). Proteins were separated on Any-KD PAGE gels (Biorad, USA) and transferred to PVDF membranes (Millipore, USA). Membranes were blocked and incubated overnight at 4°C with primary antibodies. After rinsing, the membranes were incubated with secondary antibodies for 30 min at room temperature, rinsed, and developed using chemiluminescence (Cell Signaling Technology, USA). Primary antibodies used: anti-MyoVa (LF-18; Sigma), anti-MyoVa (3402S, Cell signaling), anti-N-Cadherin (SC-7939; Santa Cruz Biotech), Gnb5 (ab72406; Abcam). For detection HRP-conjugated secondary antibodies were used (Cell Signaling Technology, USA).

### RT-qPCR

Total RNA was extracted using Direct-zol RNA Miniprep Kits (Zymo Research, USA), according to the manufacturer’s protocols. For tissue, injected animals with AAV coding for FL1 were sacrificed and infected brain tissue was quickly extracted in ice-cold PBS using a fluorescent dissecting scope. 25 mg of brain tissue was used for RNA extraction. 400 ng of total RNA was used for reverse transcription using LunaScript™ RT SuperMix Kit (NEB, USA). qPCR was performed using Forget-Me-Not™ EvaGreen® qPCR Master Mix (Biotium, USA). Data are presented as relative mRNA levels of the gene of interest normalized to GAPDH mRNA levels. Primers used for quantification: gnb5 sense 5′ GGGAACAAAGTCCTGTGCAT 3′, antisense 5′ GCGTGCTCCTTGTTCGTAGT 3′; myo5a sense 5′ TCCAGAAGCGTGTCACAGAG 3′, antisense 5′ CTTCTTCCTTTGCCTTGCTG 3′; flailer sense 5′ CAAAGGCCACGGGAACAAAG 3′, antisense 5′ CCAGAGGCACCTTCTTCTCA 3′; gapdh: sense 5′ ATGGTGAAGGTCGGTGTGAA 3′, antisense 5′ CATTCTCGGCCTTGACTGTG 3′.

### Stereotactic injection into the mouse brain

For stereotactic injections into the ventral hippocampus, P28–P30 wild-type, and FL mice were deeply anesthetized by isoflurane administration delivered at 1–5%. Craniotomy was performed according to approved procedures. Seven hundred nanoliters of AAV PHP.eB mixture of FL1 (sgRNA (2.5 × 10^13^ vg/ml) + Cas9n (7 × 10^13^ vg/ml)) or tdTomato alone (3,5 × 10^13^ vg/ml) were injected at 100 nl/min into the ventral hippocampus (anterior/posterior: − 3.52; mediolateral: 2.65; dorsal/ventral: − 3). Animals were sutured and analgesia was given (Buprenex, 1 mg/kg, i.p.). Animals were allowed to fully recover, and 3–4 weeks later, behavioral experiments were conducted. For electrophysiology recording, we made use of HSV viral particles coding for sgRNA + Cas9n + mRuby3 (1 × 10^9^ infectious units/ml) or mRuby3 alone as control (1 × 10^9^ infectious units/ml). Three hundred nanoliters of HSV at 100 nl/min was injected into the ventral hippocampus at P20. Analgesia was given (Buprenex, 1 mg/kg, i.p.) after surgery. Forty-eight to 72 h later, acute brain slices were prepared.

### Intraventricular injections

Injections were performed as in [13]. Briefly, newborn wild-type and FL animals were anesthetized by hypothermia on an aluminum plate over ice maintaining the temperature above 1°C to avoid frostbite. One microliter of AAV PHP.eB mixture of FL1 (sgRNA (2.5 × 10^13^ vg/ml) + Cas9n (7 × 10^13^ vg/ml)) or tdTomato alone (3.5 × 10^13^ vg/ml) were injected into the cerebral ventricles bilaterally. Injection was performed using a 10 μl Hamilton syringe with a 32G beveled needle. The injection site was located half the distance between bregma and lambda, 1 mm lateral to each side, and 3 mm deep. After injection animals were placed on a warming pad until they recovered color and regained movement to be later returned to their home cage.

### Brain sectioning and mounting

After behaviors to assess brain infection, animals were deeply anesthetized, and half of the brain was extracted and submerged to fix for a minimum of 24 h in PBS CaMg + 4% PFA + 4% Sucrose into 30 mL flasks. After fixation, a Leica VT1000s vibratome was used to cut 100 μm coronal sections. Slices were kept in PBS and mounted using Fluoromont G (EMS, Hatfield, PA) to preserve the fluorescence signal. Brain images were captured with a Nikon Eclipse TE2000 epifluorescence microscope or a Nikon C2 + Confocal (Nikon, USA).

### Behavioral testing

For all behavioral testing, P28–P30 male and female wild-type and FL animals were injected as previously described with AAVs coding for tdTomato alone (control) or together with FL1 (Cas9n + sgRNA). 8 weeks after infection animals were used for behavioral testing as has been previously described [13, 28].

#### Grooming behavior

Animals were individually placed into a new cage and allowed to habituate for 20 min. After habituation, animals were filmed for 1 h between 19:00 and 21:00 h with a red light (2 lx) in a Behavior Spectrometer testing chamber (Behavioral Instruments). Grooming was quantified automatically and corroborated manually using the Viewer software. The total time spent grooming in a 1 h interval was determined. Grooming included all sequences of face-wiping, scratching/rubbing of head and ears, and full-body grooming.

#### Elevated plus maze test

For the elevated plus maze, open arms were illuminated by 60 lx light while the dark (closed) arms were lit by 10–20 lx. Before testing, animals were left to habituate to 10–20 lx light for 1 h. For testing, animals were placed in the center of the maze and allowed to explore the maze freely for 5 min, all movements were recorded. Analysis was performed using the automated tracking software Noldus Ethovision. Anxiety-like behavior was determined by the time spent in the open arm during the 5-min interval.

#### Light and dark test

Mice were habituated to 20–40 lx light 1 h prior to testing. Testing was conducted in a 2-chamber apparatus (Med Associates) where one side was left completely dark (dark chamber) and the other illuminated at 1000 lx (light chamber) with an overhead lamp. Mice were placed in the dark chamber and a connecting door between dark and light chambers opened. Mice were left to freely explore for 5 min. The latency to emerge to the light chamber, the number of crosses, and the total time spent in the light chamber were automatically scored by Noldus Ethovision software. Anxiety-like behavior was determined by the time spent in the light chamber, and the latency to enter it in a 5 min interval.

#### Fear conditioning

Animals were habituated to the experimenter and behavioral room 2 days previous to testing. Animals were taken to a behavioral room and placed into an isolated fear conditioning chamber (Med Associates Inc.), left to habituate for 8 min for 2 consecutive days, and returned to their home cage. At day 3 (training day), animals were placed on the same chamber and let explore freely for 2 min followed by a 2-s 0.6-mA mild shock. After shock, animals were left to explore for 3 more min and returned to their home cage. At day 4 (testing day), mice were placed in the conditioning context, and freezing to the context provided by the box alone was assessed over 5 min. Freezing behavior was recorded, and analysis was performed using FreezeFrame (Actimetrics, USA).

### Data analysis

All values are presented as mean ± standard error (SE). The number of experiments, animals, or cells is indicated in each figure legend. Data normality was checked using the Shapiro–Wilk test. Statistical analyses were performed using one-way ANOVA followed by the Bonferroni post hoc test. Values of *p* < 0.05 are considered statistically significant. *p < 0.05, **p < 0.01, ***p < 0.001. All statistical analyses were performed using GraphPad Prism.

### Supplementary Information

**Additional file 1:** **Supplementary Figure 1.** FL1 DN-CRISPRs successfully target the flailer genomic locus without targeting gnb5 or myo5a. (A) Representative sequences of PCR products sequenced to determine changes in genomic sequence at the *flailer *locus after FL1 infection. (B-C) Representative sequences of gnb5 (B) and myo5a (C) genomic locus showing no alteration in the sequences were FL1 DN-CRISPRs target the endogenous genes. (D-E) SURVEYOR assay to analyze gene editing of gnb5 (D) or myo5a (E) shows no editing at the locus of endogenous genes when Cas9n is used. Note that co-infection of FL1 and Cas9 produces double strand breaks and editing of gnb5 and myo5a.

**Additional file 2:** **Video 1.** Intracerebroventricular injection of FL1 recovers seizures in FL mice. Recording of FL and FLicv animals in their home cage exhibiting the recovery of seizure episodes in animals treated with DN-CRISPRs.

**Additional file 3.** Raw data.

## Data Availability

All data generated or analyzed during this study are included in this published article and its supplementary information files.

## Abbreviations

AAV: Adeno-associated virus
ACSF: Artificial cerebrospinal fluid
AMPA: α-Amino-3-hydroxy-5-methyl-4-isoxazolepropionic acid
ASD: Autism spectrum disorder
CNS: Central nervous system
CRISPR: Clustered regularly interspaced short palindromic repeats
DN-CRISPRs: Double-nicking CRISPRs
DIV: Days in vitro
EPSC: Excitatory post-synaptic currents
KMT2C: Lysine N-methyltransferase 2C
LTD: Long-term depression
LTP: Long-term potentiation
PP-LFS: Paired-pulse low-frequency stimulation
