# Supplementary figures and images for: Removal of a partial genomic duplication restores synaptic transmission and behavior in the MyosinVA mutant mouse Flailer
Source: BMC Biol. 2023 Nov 14;21:232. doi: 10.1186/s12915-023-01714-y (PMC10644554; doi:10.1186/s12915-023-01714-y)

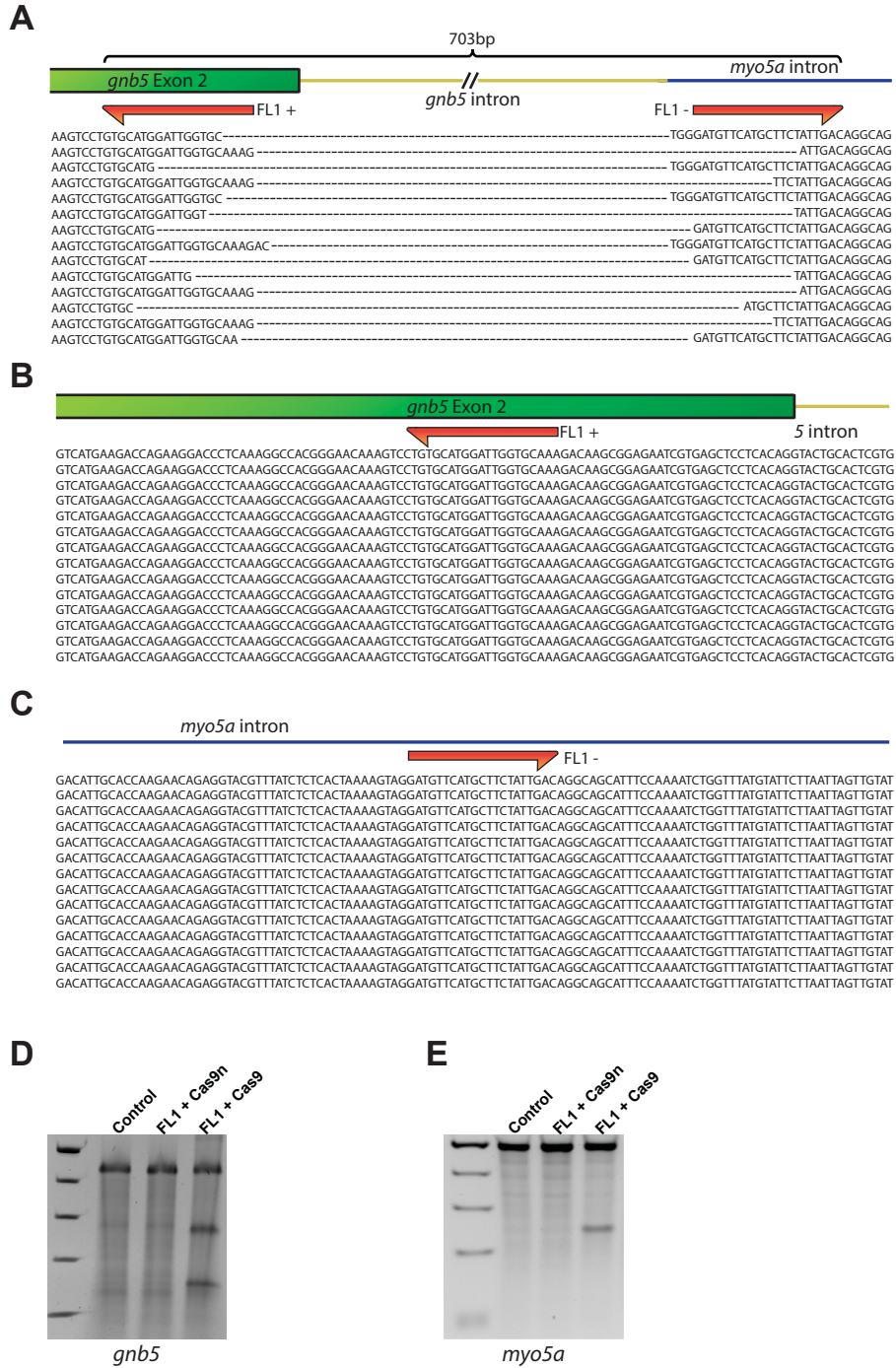

Supplementary Figure 1. BUSTOS et al.

Supplement: Supplementary file 1 — Additional file 1: Supplementary Figure 1. FL1 DN-CRISPRs successfully target the flailer genomic locus without targeting gnb5 or myo5a. (A) Representative sequences of PCR products sequenced to determine changes in genomic sequence at the flailer locus after FL1 infection. (B-C) Representative sequences of gnb5 (B) and myo5a (C) genomic locus showing no alteration in the sequences were FL1 DN-CRISPRs target the endogenous genes. (D-E) SURVEYOR assay to analyze gene editing of gnb5 (D) or myo5a (E) shows no editing at the locus of endogenous genes when Cas9n is used. Note that co-infection of FL1 and Cas9 produces double strand breaks and editing of gnb5 and myo5a. [file 12915_2023_1714_MOESM1_ESM.pdf]
